# A motivation-based theoretical framework for understanding short-form video use and mental health among university students

**DOI:** 10.3389/fpsyg.2026.1890073

**Published:** 2026-07-10

**Authors:** Zikang Yan, Wei Zhang

**Affiliations:** 1 School of Materials Science and Engineering, Sichuan University of Science & Engineering, Zigong, China; 2 School of Mathematics and Statistics, Sichuan University of Science & Engineering, Zigong, China

**Keywords:** algorithmic feedback, mental health, motivational drift, self-regulation, short-form video, university students, usage motivation

## Abstract

Short-form video platforms have become a central psychological environment in university life, yet their mental health significance cannot be explained by total screen time alone. This hypothesis-and-theory article develops the Motivation-Affordance-Capacity-Outcome framework (MACO) to explain why similar short-form video duration may produce protective, neutral, problematic, or clinically meaningful outcomes among university students. MACO is revised here as a shorter, more testable, and platform-specific framework. Its distinctive contribution rests on three mechanisms: session-in-context analysis, motivational drift, and algorithmic feedback loops. The framework argues that entry motives are translated by short-form-video affordances into engagement modes; that self-regulatory capacity and baseline vulnerability shape whether use remains flexible; and that algorithmic feedback can stabilize either adaptive or maladaptive patterns over repeated sessions. The article clarifies how MACO differs from I-PACE, the active-passive model, compensatory Internet use theory, and differential susceptibility approaches by generating comparative predictions about short-form video versus long-form video, text forums, traditional television, and general social media. It also specifies falsifiable propositions, disconfirmation criteria, and operational indicators for constructs such as motivational drift, socially saturated loneliness, perceived algorithmic agency, and motivational alignment. Particular attention is given to Chinese and East Asian university contexts as theoretically important boundary conditions rather than assumed universal settings. The framework supports interventions that move beyond generic screen-time reduction toward motive-specific diagnosis, digital mindfulness, sleep-protective friction, credibility support, platform design changes, and culturally responsive mental health education.

## 1 Introduction

Research on digital media and mental health has moved beyond the simple question of whether more screen time predicts worse well-being. Aggregate duration remains useful for public communication, but it is too blunt to explain short-form video use. TikTok, Douyin, Instagram Reels, YouTube Shorts, Kuaishou, and short-video ecologies around Bilibili combine vertical full-screen immersion, algorithmic personalization, short temporal units, social metrics, and low-friction transitions. A user does not merely select content; the platform learns from watch time, pauses, replays, searches, likes, comments, follows, and skips, and then reorganizes the next exposure environment.

University students are a theoretically important population because short-form video intersects with autonomy, academic evaluation, career uncertainty, identity exploration, peer reorganization, and weakening external regulation of daily routines. A 30-minute session may represent intentional recovery after study, avoidance of academic stress, trend monitoring, metric checking after self-presentation, or goal-directed search for career and health information. These sessions can appear similar in device logs while carrying different psychological meanings.

The present article therefore develops the Motivation-Affordance-Capacity-Outcome framework, abbreviated MACO. MACO is not proposed as a replacement for existing theories of media effects or behavioral addiction. Its purpose is to arrange motivational, platform, self-regulatory, and outcome mechanisms into a short-form-video-specific framework that can explain heterogeneous findings and generate testable predictions. The revised framework emphasizes three core innovations: session-in-context analysis, motivational drift, and algorithmic feedback loops.

The article contributes in four ways. First, it shifts the analytic unit from aggregate screen time to session-in-context. Second, it specifies how MACO extends rather than merely repeats I-PACE, the active-passive model, compensatory Internet use theory, and differential susceptibility approaches. Third, it states falsifiable predictions and disconfirmation criteria so that MACO can be evaluated empirically rather than used as post-hoc descriptive language. Fourth, it treats Chinese and East Asian university contexts as boundary conditions that may modify motivational pathways, engagement modes, and intervention acceptability.

## 2 Theoretical architecture and distinctive contribution of MACO

### 2.1 From screen time to session-in-context

A session-in-context is the focal unit of MACO. It includes the emotional state before entry, the reason for entry, the time of day, the student’s immediate goal, the content encountered, the engagement mode used, the perceived ability to stop, and the consequence for the next activity. This unit is closer to the psychological process than device totals because it distinguishes a planned study break from stress avoidance, supportive community contact from comparison surveillance, and instrumental search from symptom fixation.

This shift does not imply that duration is irrelevant. Instead, duration is treated as one indicator whose meaning depends on motive, content, timing, and self-regulatory capacity. Large-scale research shows that associations between digital technology use and well-being are often small, heterogeneous, and sensitive to model specification, including among adolescents with and without mental health conditions (Przybylski and Weinstein, 2017; Orben and Przybylski, 2019; Odgers and Jensen, 2020; Fassi et al., 2025). Short-form-video-specific studies and syntheses suggest stronger concerns for attention, inhibitory control, stress, anxiety, sleep, depression, and school engagement, but they also show the need for content- and context-sensitive interpretation (Li L. et al., 2024; Zhang D. et al., 2024; Zhang L. et al., 2024; Nguyen et al., 2025; Tang et al., 2026).

Session-in-context analysis is methodologically compatible with ecological momentary assessment (EMA). EMA can capture pre-use affect, entry motive, perceived control, content category, social comparison, and post-use affect within the same day or session. By contrast, aggregate exposure models often cannot tell whether use preceded distress, followed distress, or merely co-occurred with an unmeasured vulnerability such as low self-control or academic pressure.

### 2.2 Relation to existing theories

MACO draws selectively on existing theories but gives each theory a specific function. Uses and gratifications theory explains motivational entry (Katz et al., 1973; Ruggiero, 2000). I-PACE explains how rewarding online behavior can become dysregulated through affective, cognitive, executive, and expectancy processes (Brand et al., 2016, 2019). The differential susceptibility model explains why effects vary by dispositional, developmental, and social conditions (Valkenburg and Peter, 2013). The active-passive model clarifies why engagement mode matters, including evidence that passive social media use can be linked to affective well-being, but MACO extends it by including private saving, searching, repeated watching, and anonymous participation as potentially active forms of psychological engagement (Verduyn et al., 2015, 2017, 2022; Godard and Holtzman, 2024). Compensatory Internet use theory explains distress-driven coping but does not cover the full range of entertainment, learning, self-presentation, and social participation (Kardefelt-Winther, 2014) (Table 1).

**Table 1:** MACO in relation to existing frameworks.

Framework	Primary contribution	Limitation for short-form video	MACO extension
Uses and gratifications	Specifies why users seek media: entertainment, relief, belonging, information, expression.	Needs are often treated as user-directed; algorithmic feeds can redirect needs during a session.	Treats motive as the entry point but examines how affordances and feedback transform it.
I-PACE	Explains pathways into problematic online behavior through person, affect, cognition, and execution processes.	Broad addiction framework; not specific to short-form feeds or benign and adaptive use.	Applies dysregulation logic to session-level motives, engagement modes, and feed changes.
Active-passive model	Differentiates use modes and social reciprocity.	Public activity is too narrow for short-form video; saving, replaying, searching, and private sharing also matter.	Places active/passive distinctions inside a motive-affordance-capacity sequence.
Differential susceptibility model	Explains heterogeneity through person and context.	Less explicit about vertical feed design and recommendation feedback.	Operationalizes susceptibility through capacity, timing, culture, baseline mental health, and platform type.
Compensatory Internet use	Explains online use as coping with offline distress.	Focused mainly on compensation; less suited to learning, creator, and social participation pathways.	Distinguishes adaptive recovery, avoidance, self-presentation, and information-seeking trajectories.

### 2.3 Distinctive and falsifiable predictions

Reviewer concerns about theoretical novelty are addressed by specifying what MACO predicts beyond any single source theory. First, MACO predicts that session-level motive, engagement mode, and capacity will explain post-use affect and next-task disruption beyond total duration. If duration alone predicts outcomes as well as session-in-context variables, the core premise of MACO would be weakened.

Second, MACO predicts motivational drift: the student’s initial motive for entry can diverge from later session experience and future feed composition. This prediction is not simply that distressed students use media; it is that platform feedback can redirect a bounded motive into a different motivational trajectory, such as mood repair into avoidance, information seeking into symptom fixation, or creative production into metric dependence.

Third, MACO predicts platform-specific effects. Under similar avoidance motives, short-form video should produce stronger time distortion and stopping difficulty than long-form video because clips are shorter, transitions are more frequent, and stopping cues are weaker (Yang et al., 2024). Under similar appearance-comparison motives, visually intensive short-form feeds should produce stronger body-related comparison than text-centered forums. Traditional television should show weaker personalized feedback because exposure is not updated rapidly from micro-behavior.

Fourth, MACO can be disconfirmed. The framework would be weakened if session-level motives do not improve prediction beyond duration; if engagement mode does not moderate motive-outcome associations; if feed narrowing does not predict later motive shift after controlling baseline mental health; or if short-form video affordances do not produce distinguishable patterns compared with long-form video, text forums, or traditional television.

### 2.4 Causal identification and disconfirmation criteria

A recurrent concern in recursive media-effects models is that they may become descriptively rich but empirically loose. MACO therefore treats recursion as a hypothesis about temporally ordered processes, not as a claim that every variable is always both cause and consequence. The revised framework separates four relations that future studies should estimate rather than assume: selection effects, in which students with prior distress or low self-control select into particular motives and feeds; media effects, in which platform sessions predict later affect, sleep, attention, or functioning; reciprocal effects, in which earlier outcomes reshape later motives and exposure; and third-variable explanations, in which academic stress, loneliness, personality, family pressure, or offline support explain both use and mental health outcomes. This separation is essential because a student who reports anxiety after late-night scrolling may have been made more anxious by the session, may have entered the session because of pre-existing anxiety, or may be experiencing both processes in a reinforcing loop.

For this reason, MACO is framed around falsifiable predictions. The framework would be weakened if session-level motives and engagement modes do not improve prediction beyond total duration; if short-form video affordances do not predict time distortion more strongly than comparable long-form or text-based media under the same motive; if algorithmic feed narrowing does not predict subsequent motive shift after controlling for baseline distress and prior preferences; or if self-regulatory capacity fails to moderate the links between vulnerable motives and functional impairment. These disconfirmation criteria are deliberately explicit. They prevent MACO from becoming a post-hoc vocabulary that can explain any finding after the fact.

This approach turns average-effect debates into conditional-effect hypotheses. Large-scale work showing small average associations between digital media and well-being does not contradict MACO; it sets the level of explanation that MACO must improve. If most short-form video sessions are neutral, average associations should be modest. The theoretically important question is whether specific session configurations, such as late-night avoidance under low regulatory capacity or appearance-focused exposure during high comparison sensitivity, show stronger and more consistent relations with impairment. Conversely, MACO should also predict protective configurations, such as intentional skill learning, credible health information, reciprocal community support, or creative production guided by competence rather than metrics.

## 3 Components of the revised MACO framework

**Figure 1:**
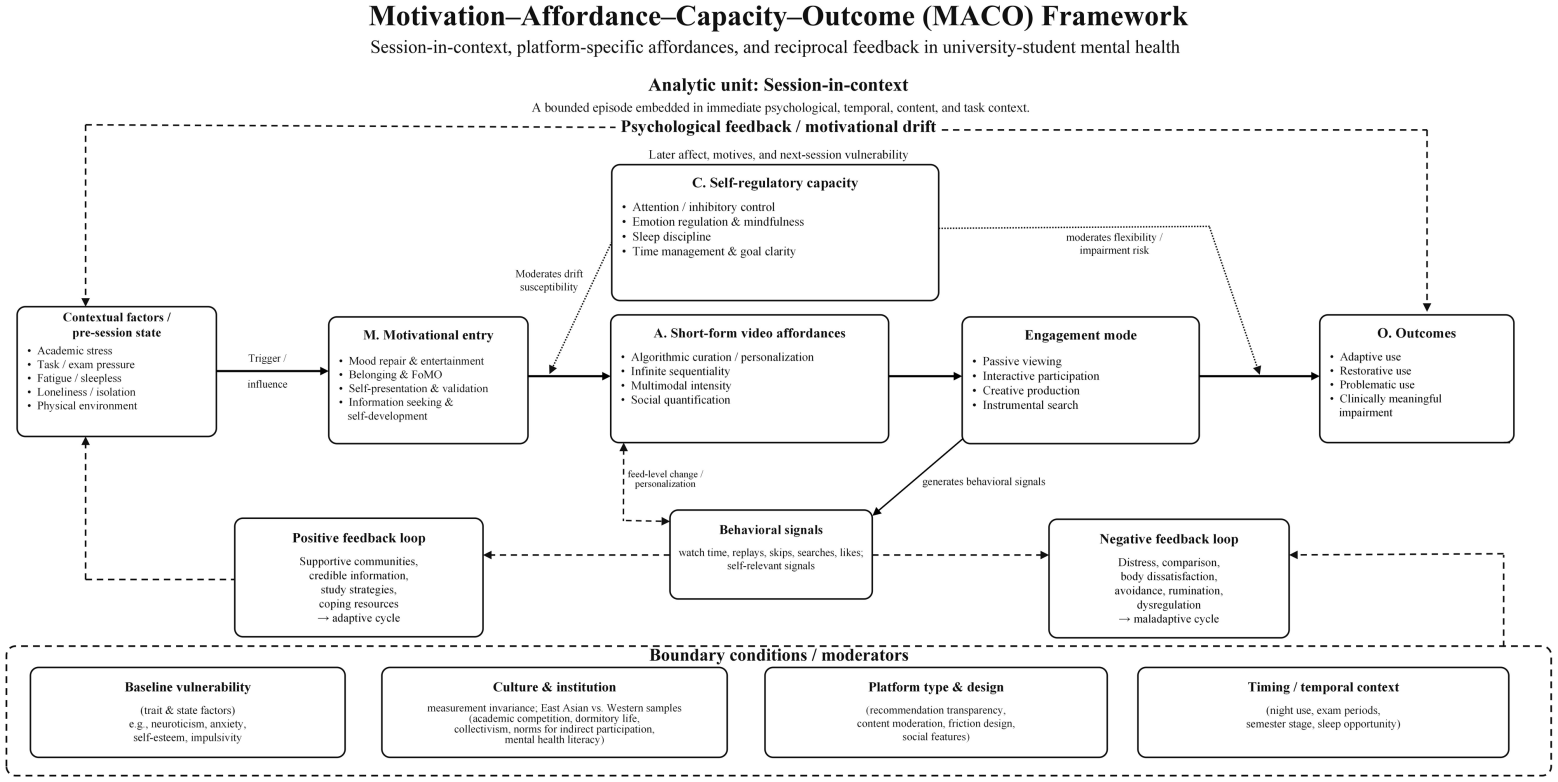
Figure 1 is a conceptual MACO diagram. It shows a session-in-context process in which contextual factors and the pre-session state trigger motivational entry, short-form video affordances shape engagement mode, and engagement leads to adaptive, restorative, problematic, or clinically meaningful outcomes. Self-regulatory capacity moderates drift susceptibility and the relation between engagement and outcomes. Behavioral signals such as watch time, replays, skips, searches, and likes update algorithmic personalization. Positive feedback loops can support credible information and adaptive use, while negative feedback loops can reinforce distress, comparison, avoidance, rumination, and dysregulation. Baseline vulnerability, culture, platform design, and timing operate as boundary conditions.

Figure 1 summarizes the MACO process and its feedback loops.

### 3.1 Motivational entry and engagement modes

MACO specifies four entry motives: mood repair and entertainment, social belonging and fear of missing out (FoMO), self-presentation and status validation, and information seeking and self-development. These motives are profiles rather than mutually exclusive categories, and they are consistent with short-form-video-specific motivation research in Chinese platform settings (Dong and Xie, 2024). They are enacted through passive viewing, interactive participation, creative production, and instrumental search. A passive-looking behavior may still be active psychologically when it involves deliberate saving, repeated watching, anonymous observation, or comparison monitoring.

### 3.2 Short-form video affordances and platform specificity

Four affordances are central to short-form video: algorithmic curation, infinite sequentiality, multimodal intensity, and social quantification. These affordances are not unique in isolation, but their combination creates a distinctive environment. Algorithmic curation rapidly personalizes exposure; infinite sequentiality reduces stopping cues; multimodal intensity compresses face, body, music, text overlay, pace, and emotional narrative; and social quantification translates recognition into metrics. Table 2 states comparative predictions that justify treating short-form video as more than a generic social-media category.

**Table 2:** Comparative predictions for short-form-video specificity.

Comparison	Shared motive	MACO prediction	Disconfirmation implication
Long-form video	Avoidance or mood repair	Short-form video should produce greater time distortion and stopping difficulty because transitions are more frequent and stopping cues are weaker.	If long-form and short-form video show equivalent time distortion under the same motive and capacity, the platform-specific component is weakened.
Text-centered forums	Belonging or comparison	Short-form video should intensify appearance, lifestyle, and productivity comparison when visual self-relevance is high.	If visual intensity adds no prediction beyond motive and duration, multimodal intensity is less central than MACO claims.
Traditional television	Entertainment or relief	Traditional television should show weaker motivational drift because exposure is less rapidly personalized by micro-behavior.	If traditional television produces similar feed-like drift, MACO should be broadened beyond short-form video.
General social media	Social participation	Private saving, repeated viewing, searching, and anonymous participation should count as active psychological engagement when they are intentional and self-relevant.	If public interaction alone predicts outcomes, MACO’s broader engagement-mode claim is weakened.

### 3.3 Self-regulatory capacity and outcomes

Self-regulatory capacity includes attention control, inhibitory control, emotion regulation, mindfulness, sleep discipline, time management, physical activity, and clarity of goals. Capacity varies between students and within the same student across days. Fatigue, stress, loneliness, examination pressure, interpersonal conflict, and sleep deprivation reduce capacity, making a feed that is restorative during the day more likely to become uncontrolled at night. This capacity-centered logic is consistent with evidence on response inhibition, self-control, physical activity, and short-video-related functioning among university students (Jin et al., 2026; Liu et al., 2025; Wang et al., 2025; Yang et al., 2025).

MACO distinguishes four outcome states. Adaptive use serves an endorsed goal and supports competence, connection, or credible information. Restorative use is a bounded recovery interval that helps the student return to a next task. Problematic use involves repeated loss of control, sleep delay, procrastination, guilt, comparison, or avoidance. Clinically meaningful impairment involves persistent functional disruption or distress that requires professional evaluation. This distinction avoids pathologizing all use while still identifying transitions into dysregulation.

### 3.4 Algorithmic feedback: negative and positive loops

Algorithmic feedback is retained as a core MACO mechanism, but the evidentiary status is clarified. Technical studies using data donation and algorithm audits show that TikTok recommendation traces can be collected and that viewing duration, liking, following, and other behavioral signals contribute to personalization (Zannettou et al., 2024; Vombatkere et al., 2024). These studies do not directly test psychological motives, motivational drift, or mental health mediation. Therefore, MACO treats algorithmic feedback as a theoretically plausible but currently under-tested psychological mechanism.

Feedback loops are not only risky, and their direction should be treated as an empirical question. Behavioral signals may update the feed toward supportive communities, credible health information, study strategies, creative models, or coping resources; they may also narrow exposure around distress, comparison, body dissatisfaction, productivity pressure, or avoidance. The following propositions specify how this loop logic can be tested rather than assumed.

A positive algorithmic feedback loop can be specified as a staged process rather than a general benefit claim. First, students may diversify behavioral signals by searching for credible sources, saving useful clips, following supportive or educational accounts, skipping comparison-oriented content, or using recommendation-reset tools when the feed becomes too narrow. Second, these signals may recalibrate the exposure environment toward resource-rich content, such as study strategies, coping skills, peer support, career information, or creative models. Third, if the student experiences greater perceived control and the session supports the next offline activity, later entry motives may become more aligned with recovery, learning, help seeking, or creative production rather than avoidance, comparison, or rumination. This positive loop remains a hypothesis: it should be supported only when feed diversification or reset behavior precedes measurable changes in later content exposure, perceived control, and post-session functioning.

### 3.5 Operationalizing key MACO constructs

Operationalizing key MACO constructs for future empirical testing is shown in Table 3.

**Table 3:** Operationalizing key MACO constructs for future empirical testing.

Construct	Definition	Possible dimensions	Example indicators
Session-in-context	A bounded episode of use embedded in immediate psychological, temporal, content, and task context.	Pre-use affect; entry motive; time of day; content category; engagement mode; perceived control; post-use consequence.	EMA prompt before and after use; logged session timing; next-task readiness; perceived ability to stop.
Motivational drift	A divergence between initial entry motive and later session experience or future feed composition.	Entry motive; post-use motive shift; feed-topic narrowing; repeated exposure to self-relevant themes.	Pre-use motive vs. post-use reflection; content-category sequence; perceived shift from recovery to avoidance or from learning to fixation.
Socially saturated loneliness	High exposure to social faces, voices, disclosures, or communities without durable reciprocal support.	Perceived social presence during use; offline reliable support; post-use loneliness; reciprocity.	Feeling socially surrounded during a session but lacking someone to contact after; low durable support despite high social-content exposure.
Perceived algorithmic agency	The extent to which students perceive the feed as understanding, guiding, supporting, trapping, or manipulating them.	Perceived feed control; perceived personalization; reset ability; perceived manipulation; autonomy.	Items on whether the platform feels easy to steer, hard to stop, supportive, or exploitative.
Motivational alignment	The degree to which platform use serves the student’s endorsed goals rather than replacing them.	Intended goal; session outcome; cost–benefit appraisal; next-task compatibility.	Before-use intention check; after-use assessment of whether the session met the need and helped or harmed the next activity.
Cultural and institutional boundary conditions	Contextual features that shape motive activation, engagement visibility, self-regulation, and intervention acceptability in university life.	Environment-level constraints: residence arrangement, room density, lights-out or sleep-management rules; calendar-level pressure: examination weeks and civil-service or postgraduate preparation periods; person- or session-level perceptions: peer comparison, indirect participation norms, perceived external self-regulation scaffolding.	Level 2/environment: dormitory room density, shared-room arrangement, sleep-management rules; Level 2/calendar: weeks before postgraduate or civil-service examinations; Level 1/session or person-day: perceived peer comparison in shared rooms, perceived lights-out constraint, private sharing, saving, anonymous commenting, or deliberate observation; Level 2/person: perceived access to non-stigmatizing academic or psychological support. Cross-cultural validation should test measurement invariance for motivational drift, engagement mode, perceived algorithmic agency, and motivational alignment before comparing path strengths across cultural groups.

## 4 Motivational pathways and transition points

The four pathways are retained but condensed to reduce redundancy. Each pathway can be adaptive, restorative, problematic, or clinically relevant depending on motive, content, capacity, timing, and feedback (Table 4).

**Table 4:** Condensed motivational pathways and expected transition points.

Pathway	Adaptive/restorative form	Risk transition	Likely outcomes and tests
Mood repair and entertainment	Intentional, bounded humor or relaxation that supports recovery and return to study.	Relief becomes avoidance; flow and infinite scrolling delay sleep or academic tasks.	Procrastination, sleep delay, time distortion, guilt; test with EMA and experimental stopping-cue designs (Roberts and David, 2023; Xie et al., 2023; Li G. et al., 2024).
Belonging, FoMO, and comparison	Reciprocal warmth, community discovery, peer connection, and stigma reduction.	Trend monitoring becomes FoMO or comparison surveillance; social feeds feel full but offline support remains weak.	Loneliness, envy, comparison, social anxiety; test reciprocity, warmth, and durable support (Godard and Holtzman, 2024; Zhao and Kou, 2024).
Self-presentation and validation	Creative production supports autonomy, competence, digital skills, and portfolios.	Expression becomes metric dependence; self-worth becomes tied to likes, views, or reach.	Anxiety, contingent self-worth, checking; test authenticity, metric visibility, and validation contingency (Lee et al., 2020).
Information seeking and self-development	Credible, actionable content supports competence, help seeking, and learning.	Search drifts into symptom fixation, misinformation, body comparison, or commercialized insecurity.	Health anxiety, body dissatisfaction, help seeking, or self-efficacy; test credibility, topic, and comparison intensity (Minadeo and Pope, 2022; Blackburn and Hogg, 2024).

### 4.1 Mood repair and entertainment

Mood repair is the most ordinary but theoretically important entry point into short-form video use. In adaptive form, students use humorous, aesthetic, musical, or relaxing clips as a bounded recovery interval between demanding tasks. The session has a recognizable beginning and ending, and the student returns to study, sleep, or social interaction with more energy. This form of use should not be pathologized. It may support recovery in the same way that a short walk, conversation, or music break can support emotional regulation. MACO therefore distinguishes restorative mood repair from avoidant mood repair rather than treating entertainment as inherently trivial or harmful.

The risk transition occurs when relief becomes the main way of avoiding an aversive task or emotion. Examination stress, unclear assignments, interpersonal discomfort, loneliness, and fear of failure can make academic work feel threatening. Short-form video offers immediate relief, and relief is strongly reinforcing. Because infinite sequentiality removes stopping points and multimodal intensity absorbs attention, the student may continue watching beyond the initial intention. The key empirical indicators are pre-use stress, stated intention, time distortion, perceived inability to stop, and next-task readiness. A 10-minute restorative break and a 10-minute avoidant episode may be identical in logs but different in psychological function. This is why MACO locates the transition at the session level rather than in total weekly duration.

### 4.2 Social belonging, FoMO, and comparison

The social pathway is similarly double-edged. Short-form video can support belonging by giving students access to shared jokes, campus trends, peer culture, identity communities, and practical advice. For students who are socially isolated, international, commuter-based, or hesitant to disclose distress offline, algorithmically discovered communities may reduce shame and provide language for experience. Active engagement can include public comments, direct sharing, private circulation, saving, repeated viewing, or group watching in dormitories. In East Asian contexts, these less visible forms of participation may be especially important because social presence does not always require public self-display.

The risk transition occurs when belonging becomes surveillance. A student may monitor trends not because they are enjoyable but because missing them feels socially costly. FoMO, comparison, and loneliness can then interact; this comparison logic is rooted in classic social comparison theory (Festinger, 1954). A feed full of bodies, relationships, academic productivity, travel, consumption, or creator success may make students feel socially surrounded but not socially supported. MACO uses the term socially saturated loneliness to describe this condition: the student encounters abundant faces, voices, and disclosures, yet lacks dependable reciprocal connection. Future studies should therefore distinguish momentary social presence during use from durable support after use. A session may reduce loneliness while scrolling but leave the student no more supported when a real problem appears.

### 4.3 Self-presentation and status validation

Self-presentation is not inherently maladaptive. Creative production can support autonomy, competence, identity coherence, digital skill, and career-relevant portfolios, consistent with self-determination theory’s emphasis on autonomy and competence (Deci and Ryan, 2000). Students may use short videos to explain academic knowledge, document art, share laboratory practice, teach language, present public-service activities, or explore professional identities. These activities can be developmentally meaningful, especially when production is guided by contribution, creativity, or skill formation. For this reason, MACO distinguishes expressive production from evaluative production. Expressive production is organized around meaning and competence; evaluative production is organized around anticipated metrics, algorithmic reach, and audience approval.

The risk transition occurs when platform metrics become the dominant standard of self-evaluation. Likes, views, comments, shares, followers, and recommendation reach may initially provide feedback for learning, but they can also create contingent self-worth. Students may interpret weak engagement as rejection or incompetence, or unexpectedly strong engagement as pressure to repeat a style that the algorithm rewards. In creator-oriented student groups, the boundary between career development and metric dependence can become blurred. MACO predicts that self-presentation will be protective when authenticity and competence are high, but more strongly associated with anxiety, compulsive checking, and self-objectification when validation contingency and metric monitoring dominate.

### 4.4 Information seeking and self-development

Information seeking is increasingly central because many students treat TikTok, Douyin, Bilibili, and YouTube Shorts as informal search engines. They use short videos for study strategies, software tutorials, fitness guidance, mental health explanations, financial tips, career planning, and examination preparation. In protective form, this pathway lowers access barriers. A student reluctant to visit a counseling center may first encounter language for anxiety through a short video; a student confused about a course may find a clear explanation; a student with limited social capital may discover career possibilities. These benefits should be acknowledged because they explain why simple prohibition is unconvincing to students.

The risk transition is epistemic and affective. Short-form video rewards clarity, confidence, emotional resonance, and repetition, but these qualities are not equivalent to accuracy. Health, body-image, finance, and career content can become misleading when popularity is treated as expertise. Mental health clips can support help-seeking, but repeated exposure to symptom narratives may encourage over-identification or rumination. Fitness clips can promote activity, but the feed can drift toward appearance surveillance or body dissatisfaction. MACO therefore treats information seeking as conditionally beneficial: it is adaptive when content is credible, contextualized, actionable, and non-comparison-based; it becomes risky when inquiry is transformed into fixation, misinformation, commercialized insecurity, or narrow self-optimization.

### 4.5 Transition points across pathways

Across all four pathways, the decisive issue is not the motive alone but the transition point at which a motive loses alignment with the student’s endorsed goal. Mood repair becomes maladaptive when recovery turns into avoidance; belonging becomes maladaptive when participation turns into surveillance; self-presentation becomes maladaptive when expression turns into metric dependence; information seeking becomes maladaptive when learning turns into fixation. These transition points provide a parsimonious way to retain the richness of the four pathways without multiplying constructs indefinitely. They also make intervention more precise. Counselors, educators, and researchers can ask whether the session met the need that brought the student to the platform, whether the student retained the ability to stop, and whether the next offline activity became easier or harder after use.

This transition-focused approach also provides a balanced answer to risk-oriented interpretations. Heavy short-form video use is not automatically harmful, and low use is not automatically healthy. A student may use platforms extensively for creative work, learning, or social connection without clinically meaningful impairment. Conversely, a short nighttime session may be harmful if it occurs after an intention to sleep, triggers comparison, or trains the feed toward distress. This prediction is consistent with studies linking short-video overuse or addiction to sleep quality, insomnia, and procrastination-related mechanisms (Li L. et al., 2024; Zhao and Kou, 2023; Wu and Yao, 2026). MACO therefore predicts that the same observable behavior can be neutral, adaptive, or maladaptive depending on motive alignment, content ecology, self-regulatory capacity, and timing. This claim directly addresses the small-effect literature by explaining why average associations can be weak while specific session-context combinations remain clinically or educationally meaningful.

## 5 Core propositions, organized by theoretical priority and function

The propositions are organized by theoretical priority as well as thematic function. P1-P2 are core propositions because they define the distinctive session-in-context and platform-specific claims of MACO. P3-P5 are pathway propositions that specify how motives and engagement modes translate into outcomes. P6-P8 are conditional propositions concerning capacity, timing, and algorithmic feedback. P9-P10 are scope and applied propositions addressing cultural moderation and intervention. All propositions are framed as testable expectations, not settled empirical claims.

### 5.1 Core propositions: session-in-context and platform specificity

P1. Session-level motive, engagement mode, and self-regulatory capacity will predict post-use affect and next-task disruption beyond total duration.

P2. Under comparable avoidance motives and baseline capacity, short-form video will produce stronger time distortion and stopping difficulty than long-form video or traditional television because of shorter clips, more frequent transitions, and weaker stopping cues.

### 5.2 Pathway propositions: motivation, engagement, and outcomes

P3. Passive viewing will predict negative outcomes primarily when combined with vulnerable motives such as avoidance, loneliness, FoMO, or appearance comparison; passive viewing in supportive or educational content ecologies may be neutral or beneficial.

P4. Self-presentation will predict competence and well-being when production supports autonomy and authenticity, but anxiety and checking when metric dependence is high.

P5. Information seeking will predict positive outcomes when credibility and actionable guidance are high, but negative outcomes when search drifts into symptom fixation, misinformation, body comparison, or extreme self-optimization.

### 5.3 Conditional propositions: capacity, timing, and feedback

P6. Self-regulatory capacity will moderate all motivational pathways, with low-capacity days strengthening links between vulnerable motives and problematic outcomes.

P7. Algorithmic feedback will predict either motivational drift or sustained motivational alignment to the extent that feed-level changes follow earlier self-relevant engagement and remain associated with later motive shift, perceived control, or post-session functioning after controlling for baseline mental health.

Testing this proposition requires triangulation among data donation or platform archives, content-category coding, and session-level subjective reports. Causal identification will remain limited where platform data are incomplete, proprietary, or ethically unavailable for experimental manipulation, and these limits should be stated explicitly in future empirical tests.

P8. Nighttime short-form video use will show stronger associations with next-day affect, attention, sleep quality, and academic functioning than daytime use when it occurs after an intention to sleep.

### 5.4 Scope and applied propositions: culture and intervention

P9. In Chinese and East Asian university contexts, dormitory co-presence, lights-out or sleep-management rules, postgraduate and civil-service examination pressure, indirect participation norms, and external self-regulation scaffolds in shared university settings will moderate how active and passive short-form video use relates to motivational drift, perceived control, and mental health outcomes; cross-cultural measurement invariance should be tested rather than assumed.

P10. Motive-matched interventions will outperform generic screen-time reduction when the intervention targets the function that maintains use, such as avoidance, loneliness, metric dependence, misinformation, or sleep vulnerability.

### 5.5 Neutral use, beneficial use, and alternative explanations

These propositions do not make MACO a risk-only account. Negative pathways such as avoidance, comparison, metric dependence, and symptom fixation remain central because they are clinically and educationally consequential, but they do not exhaust the model. MACO also predicts neutral sessions and resource-stabilizing sessions, especially when students repeatedly encounter credible study content, supportive peer communities, or coping strategies while retaining perceived control over the feed.

Alternative explanations must also be taken seriously. Low self-control, academic stress, loneliness, depressive symptoms, social anxiety, or unstable sleep routines may explain both heavy short-form video use and poorer mental health outcomes. For this reason, MACO does not interpret every association as a media effect. It requires designs that separate baseline vulnerability from session-level exposure and later outcome. The framework’s value lies in specifying when media use should add explanatory power beyond these third variables. If baseline self-control or prior distress fully accounts for motive, engagement mode, feed exposure, and outcome, then MACO’s platform-specific contribution would be reduced.

A balanced theory must not assume that short-form video is harmful by default. Large-scale studies of digital media and well-being have repeatedly shown that average associations can be small, heterogeneous, and sensitive to analytic choices. MACO incorporates this evidence by treating neutral and beneficial use as expected outcomes under particular conditions rather than as exceptions to a risk model. A session may be neutral when it is brief, emotionally mild, and unrelated to the student’s next task. It may be beneficial when it provides credible information, social support, cultural participation, creative mastery, or emotional recovery without undermining sleep, attention, or academic responsibilities.

## 6 Causal testability and research designs

Section 2.4 outlined the causal ambiguity that MACO must confront. This section translates that logic into research designs. The key task is to separate selection effects, media effects, reciprocal effects, and third-variable explanations while preserving the session-in-context unit of analysis.

### 6.1 Designs for separating selection, influence, and reciprocity

Multi-wave panel designs can test whether baseline distress, low self-control, loneliness, or academic pressure predicts later vulnerable motives, whether vulnerable motives predict subsequent outcomes, and whether outcomes then reshape later motive or feed exposure. Random-intercept cross-lagged panel models are especially useful because they separate stable between-person vulnerability from within-person change.

The EMA designs can provide finer temporal ordering by measuring emotion before entry, session characteristics during use, and affect or task readiness after use. Data-donation studies can add feed-level exposure, repeated viewing, skipping, liking, searching, and content categories, but they should be linked with subjective reports of motive, perceived control, and post-session meaning. Content annotation and ethically constrained natural experiments can then test whether feed narrowing, recommendation reset, or stopping-cue changes alter motivational alignment.

## 7 Cultural and institutional boundary conditions

MACO is proposed as a general framework for algorithm-driven short-form video use among university students, while Chinese and East Asian contexts are treated as theoretically important boundary conditions and test cases rather than assumed universal settings. Cross-cultural invariance should therefore be tested rather than presumed.

In Chinese and East Asian university contexts, academic competition, family-linked achievement expectations, graduate-school or civil-service examination pressure, dormitory life, and indirect participation norms may intensify some pathways. Mood repair may more easily become avoidance during examination preparation. Face concerns and modesty norms may change the meaning of public posting. Private sharing, saving, anonymous commenting, deliberate observation, and group viewing may function as active engagement even when they appear passive in Western measurement instruments. These conditions should be treated as measurable moderators, such as weeks before major examinations, dormitory co-presence and co-viewing, perceived lights-out constraints, peer comparison in shared rooms, and availability of class-based or student-affairs support.

Institutional conditions also matter. Universities with accessible counseling, digital literacy courses, sleep health education, academic advising, credible platform-based health communication, and peer-support structures may buffer risks. Students without such resources may rely more heavily on platforms for support and advice, increasing vulnerability to misinformation and feed narrowing. Thus culture and institution are modeled as moderators of motive, engagement, capacity, and outcome.

Future cross-cultural tests should first examine measurement invariance for motivational drift, engagement mode, perceived algorithmic agency, and motivational alignment across Chinese, other East Asian, and Western university samples. If MACO captures a broadly transferable session-level mechanism, the associations among entry motive, engagement mode, and post-use consequence should remain structurally comparable after controlling for platform type. If culture operates as a strong boundary condition, avoidance-driven entry and indirect engagement should show stronger associations with problematic use and perceived loss of control in high-pressure East Asian university contexts than in Western samples.

## 8 Methodological recommendations

Testing MACO requires designs that capture time, motive, content, capacity, and feedback while minimizing participant burden. Cross-sectional surveys can map associations but cannot distinguish selection from influence. Longitudinal designs should measure baseline mental health, motives, engagement modes, self-regulation, physical activity, content exposure, and outcomes across at least three waves when reciprocal processes are central. EMA can capture pre-use emotion, entry motive, content type, perceived control, social comparison, and post-use affect, but full pre- and post-session surveys should not be triggered for every short-form video episode.

Trace data and data donation are useful but insufficient alone because logs do not reveal motive or meaning. Rewatching may indicate enjoyment, confusion, attraction, fear, or concern. Liking may signal approval, politeness, saving for later, or algorithm training. Future studies should therefore combine passive trace measures with low-burden subjective reports. Micro-EMA designs can restrict each prompt to one or two core items, such as entry motive or next-task readiness, whereas burst designs can concentrate intensive sampling during theoretically high-risk periods such as examination weeks, late-night windows, or the transition into university. Researchers should also report and reduce measurement reactivity, because repeated prompts may themselves increase self-monitoring and change short-form video behavior.

### 8.1 Measurement matrix and preregistered tests

To make MACO empirically cumulative, future studies should treat each proposition as a preregistered test rather than as a broad interpretive claim. A basic measurement matrix would include five columns: pre-session state, entry motive, platform affordance exposure, engagement mode, and post-session outcome. Pre-session state should include mood, stress, loneliness, fatigue, sleep intention, academic pressure, baseline vulnerability, and immediate contextual factors such as dormitory co-presence or examination preparation. Entry motive should distinguish mood repair, belonging, self-presentation, and information seeking, while allowing students to report mixed motives. Affordance exposure should include personalized recommendation intensity, clip length, visible metrics, stopping cues, and content repetition. Engagement mode should distinguish passive viewing, interaction, production, search, saving, private sharing, anonymous observation, group viewing, and repeated watching. Outcomes should be separated into immediate affect, perceived control, sleep delay, task displacement, comparison, help-seeking intention, and symptom change.

This matrix also clarifies how technical data and subjective data should be combined. App logs can identify timing, duration, switching, repeated viewing, and some forms of engagement, but they cannot reveal whether a replay reflects enjoyment, fear, attraction, concern, confusion, or diagnostic searching. EMA can reveal motive and meaning, but it is vulnerable to recall bias, participant burden, and reactivity. Content annotation can classify topics and credibility, but it cannot infer personal relevance without self-report. Data donation can improve ecological validity, but it requires strong privacy safeguards and careful minimization. The strongest designs will align high-frequency log data with lower-frequency micro-EMA reports through predefined time windows, rather than requiring complete questionnaires around every session.

For context-specific indicators, especially those derived from Chinese and East Asian university settings, future studies should treat Table 3 as an item-generation matrix rather than a finished scale. Items on perceived dormitory co-presence, lights-out or sleep-management constraints, examination and civil-service preparation pressure, class-based peer comparison, indirect participation norms, and perceived external self-regulation scaffolding should first be generated from interviews, diary entries, and brief EMA prompts. These items should then be refined through cognitive interviews, piloted with exploratory factor analysis, and validated with confirmatory factor analysis, internal-consistency estimates, test–retest reliability where appropriate, convergent and discriminant validity tests, and measurement-invariance checks across gender, grade level, residence pattern, and cultural context. Because MACO is session-oriented, researchers should distinguish Level 1 session or person-day variables such as entry motive, perceived control, and momentary peer comparison from Level 2 person or environment variables such as dormitory room density, residence arrangement, lights-out rules, and examination-period timing. Multilevel modeling should be used when these levels are combined, and multilevel reliability should be reported for repeated EMA observations.

Preregistration should specify what would count as support and non-support. For example, the motivational-drift hypothesis should be supported only if pre-use motive differs systematically from post-use experience and if feed content or recommendation narrowing predicts that shift beyond prior distress. The platform-specificity hypothesis should be supported only if short-form video produces stronger time distortion, continuation, or post-session displacement than matched long-form or text-based conditions under comparable motives. The capacity hypothesis should be supported only if attention, inhibition, mindfulness, sleep debt, or physical activity moderate pathway strength. These criteria protect the framework from confirmation bias and answer the concern that recursive media models can explain any outcome after the fact.

## 9 Intervention framework

The intervention section has been reorganized as a multi-level framework rather than a list of ideas. The central principle is motivational diagnosis: an intervention should target the function that maintains use rather than total duration alone (Table 5).

**Table 5:** Multi-level intervention framework derived from MACO.

Level	Target problem	Example strategies	Expected mechanism
Student level	Automatic or avoidant entry	Digital mindfulness: before-use intention check, during-use awareness cue, after-use reflection.	Makes motive visible and supports motivational alignment.
Student level	Nighttime dysregulation	Sleep-protective friction: scheduled downtime, grayscale mode, charging away from bed, bedtime prompts, session summaries.	Adds stopping cues when fatigue lowers capacity.
Student level	Low offline regulatory capacity	Physical activity and offline routines: evening walks, peer exercise appointments, movement breaks, study-group routines.	Provides embodied reward, mood regulation, and competing non-algorithmic reinforcement.
Institutional / shared-environment level	Vulnerable entry, weak offline capacity, and affordance-driven drift	External self-regulation scaffolds: motive-specific academic scaffolding for avoidance-driven entry; feed-literacy routines for recognizing recommendation narrowing and diversifying behavioral signals; peer-accountability routines in shared physical spaces; sleep-protective routines during high-risk academic periods.	Reduces avoidance-driven entry by lowering academic ambiguity before it becomes a vulnerable entry motive; interrupts affordance-driven motivational drift by helping students recognize recommendation narrowing and diversify behavioral signals; strengthens offline regulatory scaffolding by making stopping points, study routines, and sleep-protective norms socially visible; and stabilizes adaptive feedback loops without relying on administrative supervision as the primary mechanism.
Platform level	Feed narrowing and metric pressure	Recommendation reset tools, content-diversity controls, reduced metric visibility, credible health signals, optional stopping points.	Reduces vulnerability amplification and supports positive feedback loops.

### 9.1 From intervention list to intervention logic

At the student level, the intervention logic begins with motivational awareness rather than generic time reduction. Digital mindfulness can be operationalized as a before-use intention check, a during-use awareness cue, and an after-use reflection. These brief prompts ask what the student is trying to feel, avoid, know, or express, whether the session still serves that goal, and whether the session helped or harmed the next activity.

At the institutional and shared-environment level, the intervention logic should be stated as a mechanism rather than as a list of administrative service providers. Motive-specific academic scaffolding can reduce avoidance-driven entry by helping students convert ambiguous academic pressure into manageable next actions before short-form video becomes the default coping route. Feed-literacy routines can help students recognize recommendation narrowing, diversify behavioral signals, and evaluate whether the feed still serves the motive that brought them to the platform. In shared physical spaces, peer-accountability routines and sleep-protective environmental cues can provide external scaffolding for self-regulation during examination periods, late-night use, and dormitory co-presence.

At the platform level, the core target is design friction and feed agency. Recommendation reset tools, content-diversity controls, clearer explanations of recommendation logic, optional stopping points, reduced metric visibility, session summaries, and credible-source signals can reduce the asymmetry between students’ momentary vulnerabilities and highly optimized feeds.

Across levels, the intervention implication is mechanism-specific: avoidant entry requires motivational awareness and academic scaffolding; nighttime dysregulation requires sleep-protective friction; metric dependence requires validation and metric-management support; risky information seeking requires credibility and comparison literacy; and low offline regulatory capacity requires non-algorithmic routines such as physical activity, peer accountability, and study-group structures.

### 9.2 Integrated intervention framework

An integrated MACO intervention aligns need, design, and capacity. It identifies why students enter the platform, reduces affordance-driven drift, strengthens offline regulatory routines, and creates feedback environments that stabilize adaptive rather than maladaptive use. In Chinese and East Asian university contexts, this logic may be more acceptable when framed as support for attention, sleep, academic efficiency, emotional resilience, and career development rather than as a moral warning against addiction.

## 10 Limitations and future directions

This article remains a theoretical framework rather than an empirical test. Several mechanisms are under-tested in short-form video contexts, especially algorithmic feedback as a psychological mediator. The technical evidence on recommendation traces supports the feasibility of studying feed personalization, but it does not yet demonstrate motivational drift or mental health mediation. Future work should test these claims directly with ethically responsible designs.

The framework also needs cross-cultural validation. Chinese and East Asian contexts are used as theoretically important test cases, but MACO should not assume that active use, passive use, metric pressure, privacy norms, or platform literacy operate identically across cultural settings. Measurement invariance and comparative research are necessary.

Finally, the framework should be updated as platforms change. Generative artificial intelligence, livestream commerce, stronger recommendation transparency, or user-controllable feeds may alter the balance between negative and positive feedback loops.

## 11 Conclusion

Short-form video platforms are influential digital environments in contemporary university life. Their mental health significance cannot be captured by exposure time alone. Students use them for recovery, belonging, self-presentation, information, learning, escape, and identity work. These motives are translated by platform affordances into engagement patterns that can support competence and connection or undermine attention, sleep, academic functioning, body image, and emotional stability.

The revised MACO framework explains these divergent outcomes through session-in-context, motivational drift, self-regulatory capacity, algorithmic feedback, and boundary conditions. It is designed to be testable and falsifiable: its value depends on whether session-level motives, engagement modes, platform affordances, and feedback traces add explanatory power beyond duration and baseline vulnerability. For universities, clinicians, and platforms, the practical implication is not prohibition but diagnosis and design: identify the function of use, protect sleep, improve credibility, reduce metric pressure, diversify feedback, and support students in keeping short-form video aligned with their own goals.

## Data Availability

The original contributions presented in the study are included in the article/supplementary material, further inquiries can be directed to the corresponding author.
